# Bacterial cellulose membrane combined with BMSCs promotes wound healing by activating the notch signaling pathway

**DOI:** 10.3389/fsurg.2022.1027067

**Published:** 2023-01-16

**Authors:** Xiaoyang Wang, Jie Zhao, Xiaochuan Wang, Jingjuan Zhang, Yi Wang, Xinyue Wang, Shanshan Jia, Nian Shi, Meiqi Lu, Hongxia Su, Jixun Zhang, Duyin Jiang

**Affiliations:** ^1^ Department of Plastic and Burns Surgery, The Second Hospital of Shandong University, Jinan, China; ^2^ Emergency Medicine Center, The Second Hospital of Shandong University, Jinan, China; ^3^ Shandong Nameide Biotechnology Limited Company, Jinan, China

**Keywords:** bacterial cellulose, BMSCs, bioactive wound dressing, nanomaterial, notch signaling pathway, tissue repair

## Abstract

**Objective:**

The bacterial cellulose membrane (BCM) has been widely studied and applied as a new biomaterial for wound healing, but causes pain with frequent dressing changes. Local application of bone marrow mesenchymal stem cells (BMSCs) requires a niche. Furthermore, the effect and mechanism of the BCM combined with BMSCs have not been reported.

**Methods:**

Morphological and chemical identifications of BCMs were investigated by porosity analyses, scanning electron microscopy, and Fourier-transform infrared spectroscopy. Biological wound dressings (BWDs) were prepared by the BCM in combination with BMSCs. The biological effects of BWDs on human dermal fibroblast (HDF) and VEGF-A in human vascular endothelial cells (HuVECs) were detected *in vitro*, and the effect of BWDs on acute wounds in mice was detected *in vivo*. Collagen and angiogenesis were evaluated through hematoxylin–eosin staining and Masson staining. The expressions of *COL-1* and *VEGF-A* and the activation of the Notch signaling pathway *in vivo* and *in vitro* were detected by quantitative reverse-transcriptase polymerase chain reaction.

**Results:**

The BCM had a nanoscale structure and provided a partial niche for the survival and proliferation of BMSCs. BWDs were successfully prepared and regulated the biological behaviors of wound healing-related cells *in vitro* and upregulated the expressions of *COL-1* in HDF and *VEGF-A* in HuVECs. BWDs promoted wound healing by increasing collagen type I synthesis and angiogenesis in acute wounds in mice.

**Conclusions:**

BWDs prepared by the combination of nanomaterial BCMs and BMSCs facilitated acute wound healing, which may be regulated by activating the Notch signaling pathway.

## 1. Introduction

Bacterial cellulose is an extracellular polysaccharide mainly secreted by *Gluconacetobacter*. *Gluconacetobacter* is an aerobic gram-negative bacterium that can grow and produce very fine nanofibers in a liquid medium with various carbon and nitrogen sources using glucose as substrate. Nanofibers with lengths of 20–100 nm are intertwined, forming the bacterial cellulose membrane (BCM) with a three-dimensional network structure. The BCM has high mechanical strength, hydrophilicity, porosity (which allows selective permeability, adhesion of cell, and diffusion of the culture medium), and biocompatibility (1, 2). Previous studies have established that BCMs are widely used in medicine, hormone and protein release system, artificial skin, cartilage, meniscus, intervertebral disk, valve prosthesis, artificial cornea, urethra, etc (3, 4)..

Mesenchymal stem cells (MSCs) are pluripotent cells that can differentiate into various cell types, including bone, cartilage, muscle, fat, and connective tissue cells, and have broad application prospects in regenerative medicine. Extensive studies have shown the importance of MSCs in wound repair (5, 6), cardiovascular diseases (7), immune system disease (8), bone and cartilage diseases (9), and hematological malignancies (10). In this regard, bone marrow MSCs (BMSCs) are now well established because of their high yield of isolated cells with colony-forming potential, self-renewal capacity, MSC surface marker expression, and multi-lineage differentiation capacities *in vitro* (11).

In tissue engineering, BMSCs are usually used as seed cells for biological integration with biomaterials to ensure the stability of the cellular microenvironment or niche (12, 13). Several studies have suggested that BMSCs can adhere to the BCM and proliferate, and the characteristics of cells can be well maintained (14). Moreover, the BCM can induce BMSCs to produce type I collagen, which can provide mechanical stability for tissues and promote tissue healing (15). Although extensive research has been conducted on the effect of the BCM on promoting wound healing, no study has examined the use of the BCM combined with BMSCs for wound treatment. Thus, this study explored, for the first time, the effect and potential mechanism of BCM and BMSC biointegration in wound healing.

## 2. Materials and methods

### 2.1. Fourier-transform infrared (Ft-Ir) spectroscopy

BCMs provided by Nameide Biotechnology of Shandong, China, were evaluated through (FT-IR) spectra. The scan was conducted from 4000** **cm^−1^ to 500** **cm^−1^ with a resolution of 0.5** **cm^−1^ for each measurement. To ensure data credibility, we conducted three random sample extraction tests.

### 2.2. Pore size analysis

The pores of the BCM were evaluated through a porosity analyzer (PSMA-10, China). The BCM was immersed in water for 24** **h. Isobutanol and deionized water were mixed at 1:1 for 4–8** **h. The upper liquid (alcohol phase) of the mixture was taken to soak the BCM for about 510** **min. Finally, the BCM was installed on the aperture detector, the software was opened for testing, and the results were processed and analyzed. To ensure data credibility, we conducted three random sample extraction tests.

### 2.3. Scanning electron microscope (SEM)

The BCM was soaked in water for 24** **h, fixed on the test bench of the SEM (Quanta 200, USA), and observed after gold spraying. To ensure data credibility, we conducted three random sample extraction tests.

### 2.4. Cell culture and labeling

BMSCs (Cyagen, USA) derived from Balb/c mice were cultured in complete DMEM/low-glucose medium (HyClone, USA) containing 10% fetal bovine serum (Gibco, USA) and 1% 100 U/ml Penicillin-Streptomycin (Gibco). In BMSCs of P2 generation with a degree of integration up to 80% at the logarithmic growth stage, they were labeled by Cell Tracker TM CM-Dil (MKbio, China) according to the manufacturer's instructions. Specifically, CM-Dil was dissolved in 1** **mg/ml dimethyl sulfoxide as a working solution and added to the BMSCs (3** **ml of working solution per Petri dish). Subsequently, cells were incubated at 37 °C for 3** **min and then incubated for another 15** **min at 4 °C. Light should be avoided during labeling. Finally, BMSCs were washed with phosphate-buffered solution (PBS) and incubated with complete DMEM/low-glucose medium (Thermo Fisher Scientific, MA, USA). The culture medium was replaced once a day, and the subculture was used when the degree of fusion reached 80%–90%. BMSCs of the fifth–sixth generations were used for further utilization (16).

### 2.5. Detection of biocompatibility between BMSCs and BCM

BCMs (NanoMed Biotech, China) fermented by *Acetobacter xylinum* (17) were cut into round pieces (1.5** **cm in diameter) under sterile conditions and rehydrated in serum-free DMEM/low-glucose medium (containing 1% 100 U/ml Penicillin-Streptomycin) for 24** **h. The liquid on the surface of the BCM was dried and spread at the bottom of the 24-well plate (one piece per well). Subsequently, CM-Dil-labeled BMSCs were injected into the BCM by multi-point injection method at a density of 5 × 10^4^ cells/well. Then, 500 μl of serum-free DMEM/low-glucose medium was added to each well. The 24-well plate was incubated in a constant temperature incubator at 37 °C, 5% CO_2_ with saturated humidity for 24** **h. Finally, 50 μl of Cell Count Kit-8 (CCK-8, Elabscience, China) detection reagent was added to each well. The absorbance at 450** **nm (*OD_450_*) was measured by a microplate reader (Thermo Fisher Scientific) at 0** **h, 1** **h, and 2** **h.

### 2.6. Preparation of bioactive wound dressing

As described in 2.5, the BCMs were cut into round pieces (1.5** **cm in diameter) under sterile conditions and rehydrated in serum-free DMEM/low-glucose medium for 24** **h. The liquid on the surface of the BCM was dried and transferred to the 6-well plate (one piece per well). Subsequently, CM-Dil-labeled BMSCs were injected into the BCM at a density of 1 × 10^5^ cells/well. Thereafter, 2** **ml of serum-free DMEM/low-glucose medium was added to each well. The 6-well plate was incubated for 24** **h. The status of CM-Dil-labeled BMSCs on the BCM scaffolds was observed under an inverted fluorescence microscope (Nikon, Japan), and scaffolds with good activity were selected as bioactive wound dressings (BWDs) for further utilization (18).

### 2.7. Cell proliferation

As described in 2.6, the BWDs were prepared and transferred in 6-well plates, and 2** **ml of serum-free DMEM/low-glucose medium was added to each well. The 6-well plate was incubated for 24** **h. The culture medium was collected and filtered into a conditioned medium (CM) with a 0.22-μm filter and stored at −80 °C for further utilization.

To assess the effect of BWDs on wound healing, we screened two kinds of cells closely related to wound healing (i.e., human dermal fibroblasts [HDFs] and human vascular endothelial cells [HuVECs]) to detect the proliferation ability *in vitro*. Briefly, HDFs/HuVECs were seeded into 96-well plates at a density of 1 × 10^4^/well. CM and serum-free DMEM/low-glucose medium were added to the experimental group and control group, respectively, at a density of 100 μl/well. Subsequently, the 96-well plate was incubated for 24** **h. Then, 10 μl of the CCK-8 solution was added to each well and incubated at 37 °C. The absorbance at 450** **nm at 0** **h, 1** **h, and 2** **h was measured by a microplate reader. Three repeated parallel replicates were used at least every time.

### 2.8. Scratch-wound assay

The scratch-wound assay was used to detect basic cell migration parameters of CM on wound healing-related cells. HDFs/HuVECs were seeded into 6-well plates at a density of 1 × 10^5^/well, and 2** **ml of complete DMEM/low-glucose medium was added to each well. When cells reached 90% confluence, a thin **“**wound” was introduced by scratching with a 10–200-ml pipette tip. The cells shed due to streaking and were washed by PBS, and 2** **ml CM or blank medium (as a vehicle) was added to each well. At 0, 6, 12, and 24** **h, cells on the microscope were selected on field or fields of view to observe cell migration, and data were analyzed by ImageJ. Each condition was performed in triplicate (19).

### 2.9. Quantitative reverse-transcription polymerase chain reaction (qRT-PCR)

Total RNAs were extracted by animal tissue/cell total RNA extraction kit (Tiangen, China), and 500 ng total RNAs were used as templates of reverse-transcription with the oligo-dT primer for the qRT-PCR. The primers were synthesized by Sangon Biotech (China), and the sequences are listed in Table 1. *GAPDH* was used as the reference gene for calculations. The relative expression value was calculated with the following formula: *Δ*Ct = Ct (target gene)−Ct (reference gene), and the relative = 2^−*ΔΔ*Ct^.

**Table 1:** Primer sequences for quantitative reverse-transcription PCR.

Genes name	Primers sequence (5'→3')
*Jagged-1*	ATGCAGAACGTGAATGGAGAG
	GCGGGACTGATACTCCTTGAG
*Notch-1*	GATGGCCTCAATGGGTACAAG
	TCGTTGTTGTTGATGTCACAGT
*Hes-1*	TCAACACGACACCGGACAAAC
* *	ATGCCGGGAGCTATCTTTCTT
*VEGF-A*	CTGCCGTCCGATTGAGACC
* *	CCCCTCCTTGTACCACTGTC
*COL-1*	ATTGGGGACCCTTAGGCCAT
* *	GCTCCTCTTAGGGGCCACT
*GAPDH*	AGGTCGGTGTGAACGGATTTG
* *	GGGGTCGTTGATGGCAACA

### 2.10. BWDs transplanted into full-thickness skin defect model in mice

Six-week-old Balb/C male mice were randomly divided into three groups: control, BCM, and BWDs. Full-thickness skin defect models were made using a tissue punch on the back of the mice. Briefly, the mice were anesthetized by isoflurane inhalation, and the hair on the back was removed. A full-thickness skin defect model with a diameter of 15 mm was made using a tissue punch on the back of the mice. Then, the wound was covered with an aseptic dressing (control, saline gauze; BCM, and BWD). The radiation-sterilized anti-shrinkage aseptic ring (20 mm in outer diameter, 15 mm in inner diameter, and 2 mm in thickness) was sewn to the edge of each defective skin with a 4–0 mousse thread. Each silicone ring was sutured with eight sites for fixation (Figure 6A). At the end of the operation, the wound was covered with a 3 M transparent film.

The activities of mice and wound dressings were observed daily, and the dressing was removed on day 7. Wound healing was observed on days 0, 7, and 14 after the operation, and the wound healing rate was calculated and analyzed by ImageJ. The percentage wound closure was calculated as [(original wound area−wound area) / original wound area] × 100%. On days 7 and 14, the animals were killed with excessive anesthesia, and the wound samples were taken for further utilization. Each group contained five mice.

### 2.11. DAPT treatment

*γ*-Secretase inhibitor DAPT was purchased from AbMole (USA) and diluted with dimethyl sulfoxide (DMSO). In an inhibiting assay, HDFs were incubated with BWD-CM and DMSO or DAPT (5 μM) for 72 h. Then, the cells were harvested for qRT-PCR. In the *in vivo* experiments, full-thickness skin wounds in mice were treated with BWD and subcutaneous injection of 100 μl of DMSO or DAPT (10 μM).

### 2.12. Statistical analysis

Data were statistically analyzed using SPSS version 16.0 (SPSS Inc., Chicago, IL, USA). All data are presented as the mean ± SEM ($\bar{X\;}$ ± $\;S^{\bar{X}}$). Significance was determined by the analysis of variance or standard *t*-tests. *P* ≤ 0.05, *P* ≤ 0.01, and *P* ≤ 0.001, and NS indicated the absence of statistical difference.

## 3. Results

### 3.1. Morphological and chemical characterization of the BCM

#### 3.1.1. FT-IR study of the BCM

**Figure 1:**
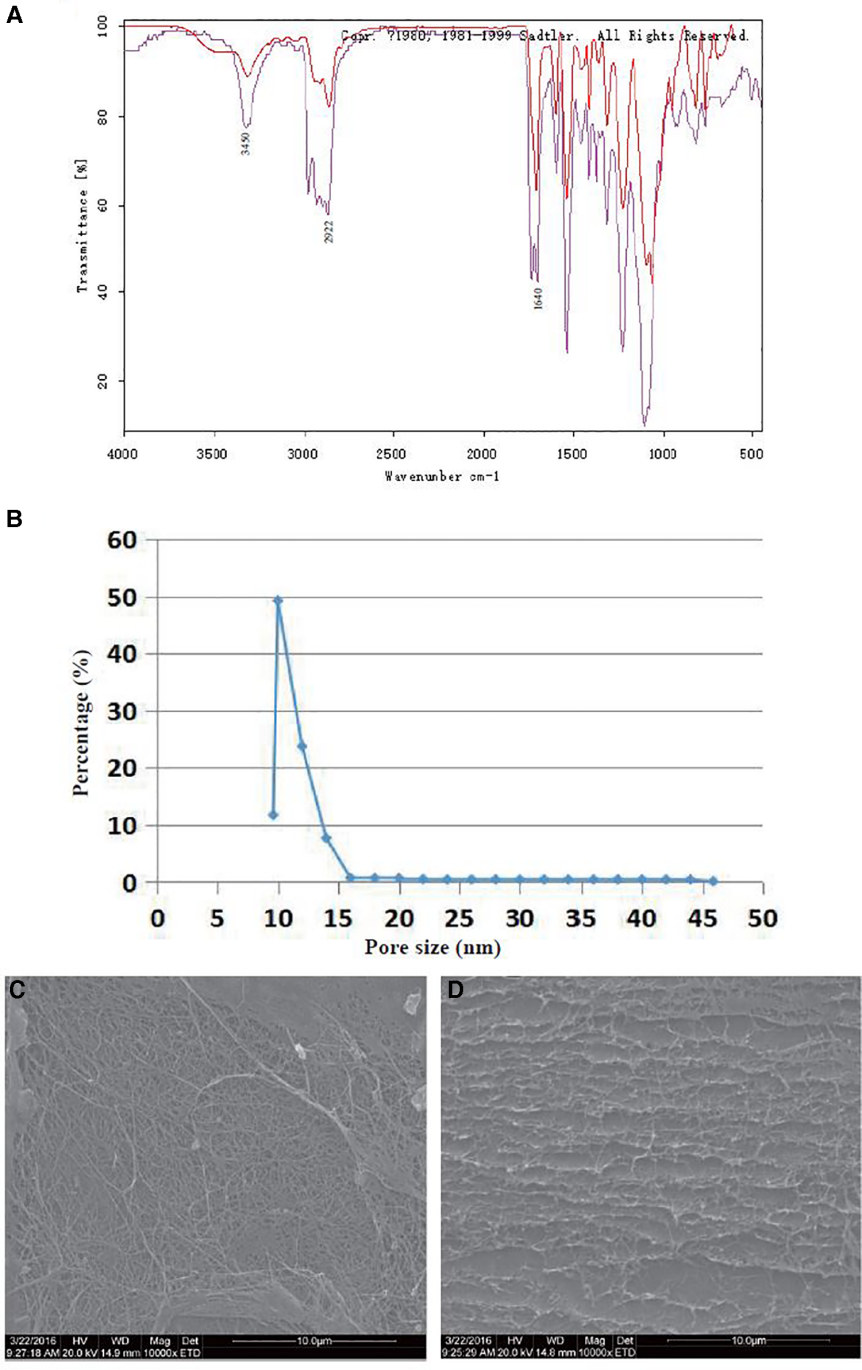
Morphological and chemical characterization of the BCM. (**A**) FT-IR spectra of the BCM: purple line, crude BCM; red line, purified BCM. (**B**) Pore size distribution of the BCM. (**C**) SEM images of the BCM surface (×10,000). (**D**) SEM images of the BCM cross-section (×10,000). (*n* = 3).

The structure of the BCM was investigated by FT-IR spectroscopy (Figure 1A). A wide and strong characteristic absorption peak that appeared at 3450 cm^−1^ was assigned to the stretching vibration of the O–H bond. The characteristic absorption bank at 2,922 cm^−1^ was due to C–H stretching, which is the characteristic absorption peak of sugars. The band at 1640 cm^−1^ is due to the stretching vibration of the carboxyl group. In this study, the FT-IR spectra of the BCM showed several bands typical for cellulose in the region from 1,500 to 1235 cm^−1^. The series of compact bands of 1200–1000 cm^−1^ indicated the stretching vibration of C–O–C of the ether bond and the stretching vibration of C–O of primary (C6) and secondary (C2 and C3) alcohols. These results were consistent with previous reports (20–22).

#### 3.1.2. Pore size analysis of the BCM

Figure 1B shows that the pore size of the BCM was <46 nm, and most sizes were distributed between 10 and 20 nm, which suggested that this kind of material was dense and had a nanoporous structure.

#### 3.1.3. SEM study of the BCM

The fiber-interleaved porous structure on the BCM surface provides structural conditions for gas transmission and bacterial isolation (Figure 1C). The layer-by-layer filtering structure composed of BCM multilayer fibers can improve its barrier performance (Figure 1D). The 3D structure of the BCM provided space for the adhesion and proliferation of BMSCs.

### 3.2. Stable proliferation of cm-dil-labeled BMSCs *in vitro*

**Figure 2:**
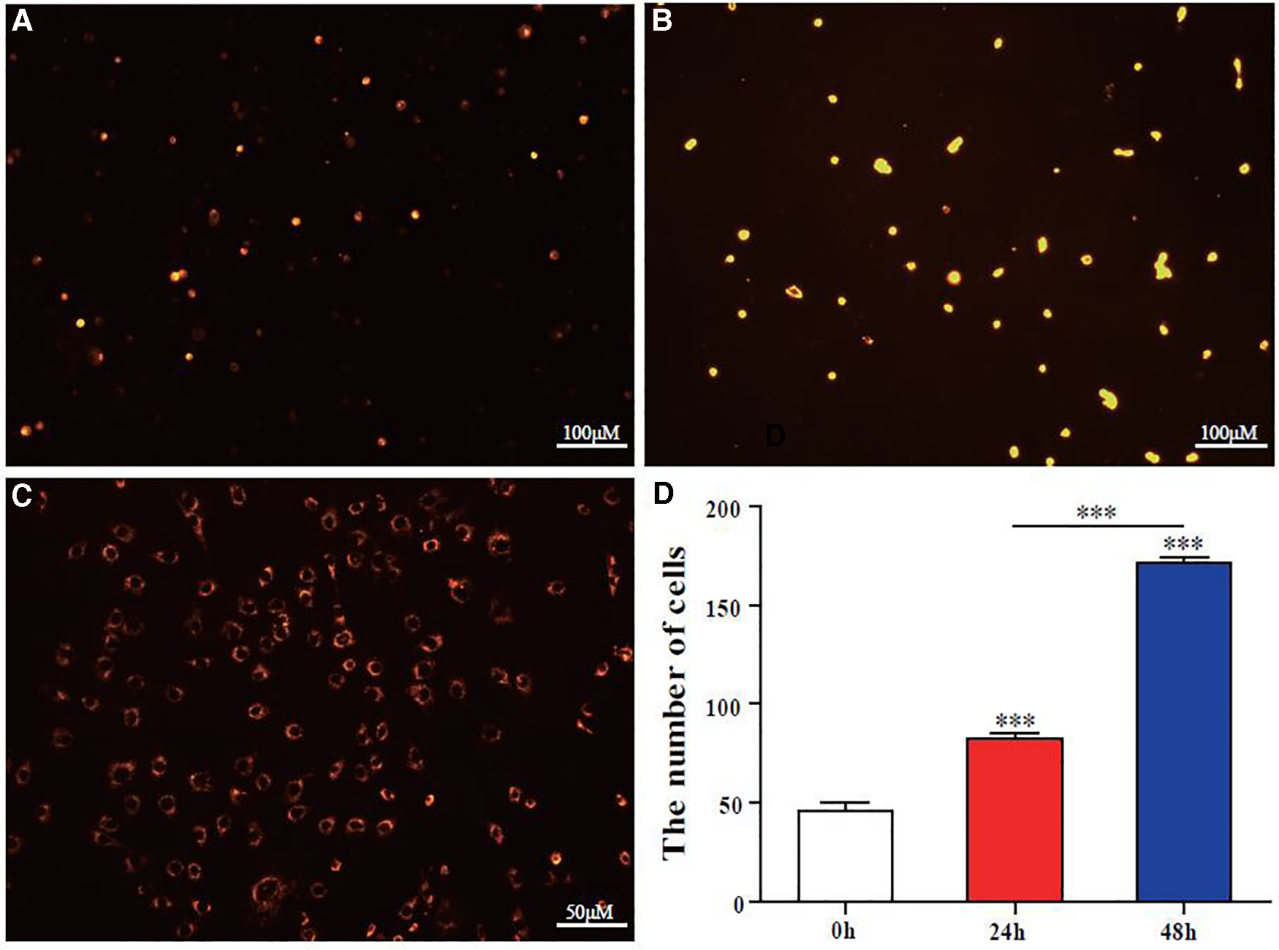
CM-Dil labeling of BMSCs and detecting proliferation *in vitro*. (**A–C**) At 0, 24, and 48** **h, BMSCs were labeled by CM-Dil (red fluorescence; bar scales = 100 μM (**A,B**) Bar scales = 50 μM (**C**)). (**D**) Quantitative analysis of BMSC proliferation in the DADM at 0, 24, and 48** **h by cell count. Error bars represent SEM (*n* = 3), *** *P* ≤ 0.001.

When CM-Dil-labeled BMSCs were observed under a fluorescence microscope, red fluorescence was observed in some labeled cells at 0** **h (Figure 2A). The number and brightness of labeled cells were significantly increased at 24** **h (Figure 2B), the labeled cells had >80% of confluence at 48** **h, and the fluorescence brightness was weakened (Figure 2C). Three visual fields were randomly selected under 10 × microscope for cell counting, and the cells labeled by CM-Dil had biological activity and could proliferate stably (Figure 2D). However, the fluorescence intensity of CM-Dil labeling decreased gradually with time. The above results provided a basis for follow-up short-term tracking of BMSCs on the BCM.

### 3.3. Preparation and biocompatibility evaluation of BWDs

**Figure 3:**
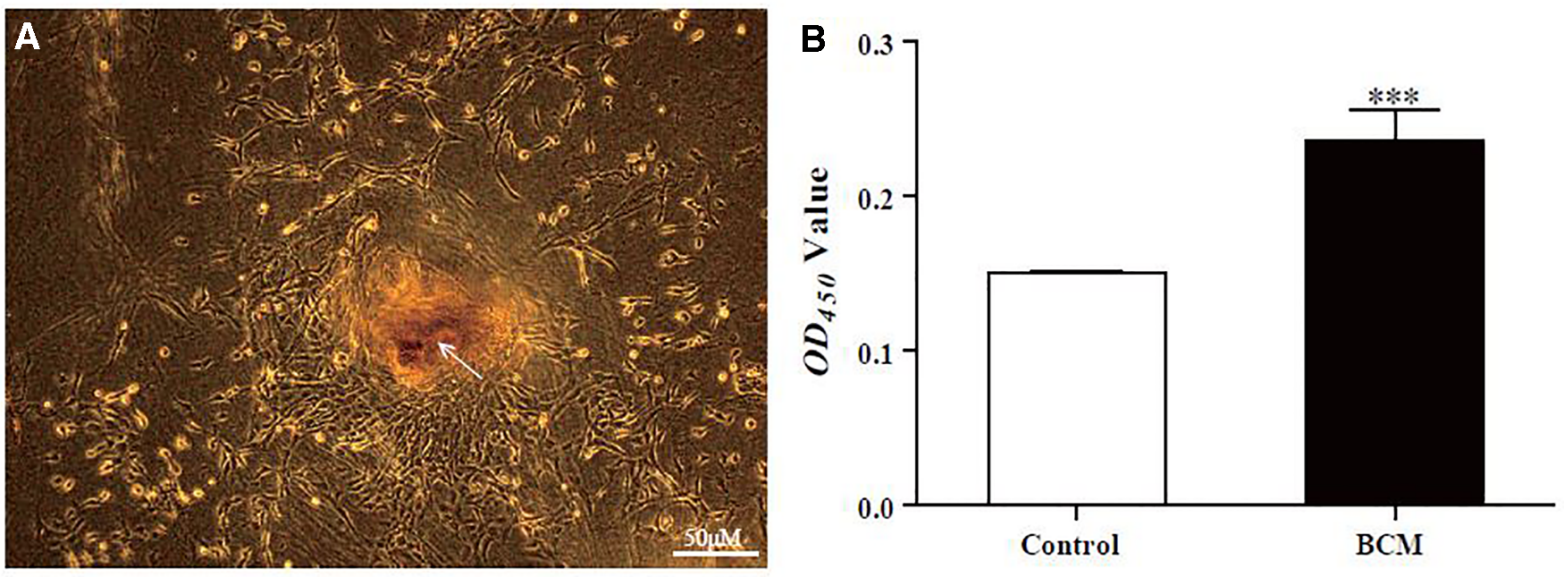
Preparation and observation of BWDs *in vitro*. (**A**) Observation of BWD loaded with BMSCs (bar scales = 100 μM; arrows, puncture point). (**B**) The proliferation of BMSCs seeded in the BCM was measured by CCK-8 (control: BMSCs without BCM intervention; BCM, BMSCs loaded on the BCM). Error bars represent SEM (*n* = 3), *** *P* ≤ 0.001.

BMSCs were injected into the BCM by the multi-point injection method to prepare BWDs. BWDs were cultured *in vitro* for 3 days and then observed under a microscope. Interestingly, in this side of the graph, BMSCs grew radially around the puncture point and adhered to the BCM (Figure 3A). On the surface and inside the BCM, adherent cells, colony cells, and apoptotic non-adherent cells were observed. In Figure 3B, quantitative analysis of CCK8 showed a significant difference (*P* < 0.001) between the two groups. These results suggested that the BCM had biocompatibility and could promote the proliferation of BMSCs; thus, it could be used to prepare bioactive dressings.

### 3.4. BWDs promote the proliferation and migration of cells related to wound repair

**Figure 4:**
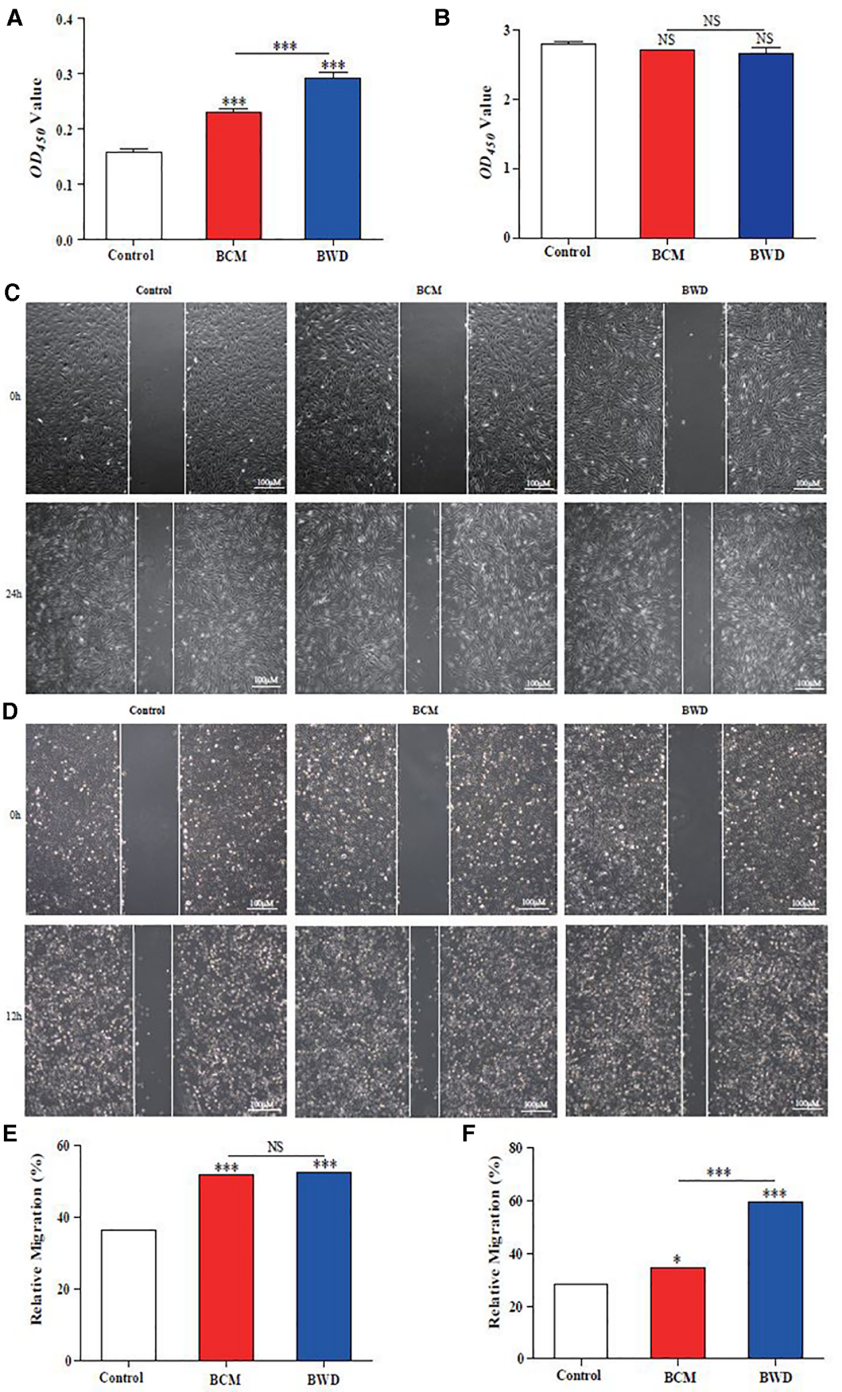
Proliferation and migration of HDFs and HuVECs. (**A**) CCK8 assay for the proliferation of HDFs. (**B**) CCK8 assay to detect the proliferation of HuVECs. (**C,E**) Scratch-wound assay for HDF migration. (**D,F**) Scratch-wound assay for HuVEC migration. Control, saline gauze; BCM, bacterial cellulose membrane; BWD, bioactive wound dressing; bar scales = 100 μM. Error bars represent SEM (*n* = 3), *P ≤ 0.05, ***P ≤ 0.001, and NS indicated no statistical difference.

Wound repair is closely related to the proliferation and migration of fibroblasts and angiogenesis. The BCM and BMSCs may play an important role in the proliferation and migration of fibroblasts and endothelial cells. To confirm this hypothesis, CM was used to treat HDFs or HuVECs. We conducted a CCK8 assay to detect cell proliferation and scratch-wound assay for migration. The BCM and BWD promoted the proliferation (Figure 4A) and migration (Figures 4C,E) of HDF at 24 h. BWD had a more significant effect on proliferation, but no difference was found in promoting cell migration. In the experimental evidence on HuVECs, the BCM and BWD had no significant effect on proliferation at 24 h (Figure 4B). However, the BCM and BWD promoted migration (Figures 4D,F) of HuVECs at 12 h, and the effect of BWD was even more significant. Taken together, these results suggested that BCMs and BWDs had good biocompatibility and could accelerate wound healing by promoting the proliferation and migration of fibroblasts and endothelial cells.

### 3.5. BWDs upregulate the expression of wound healing-related genes *in vitro*

**Figure 5:**
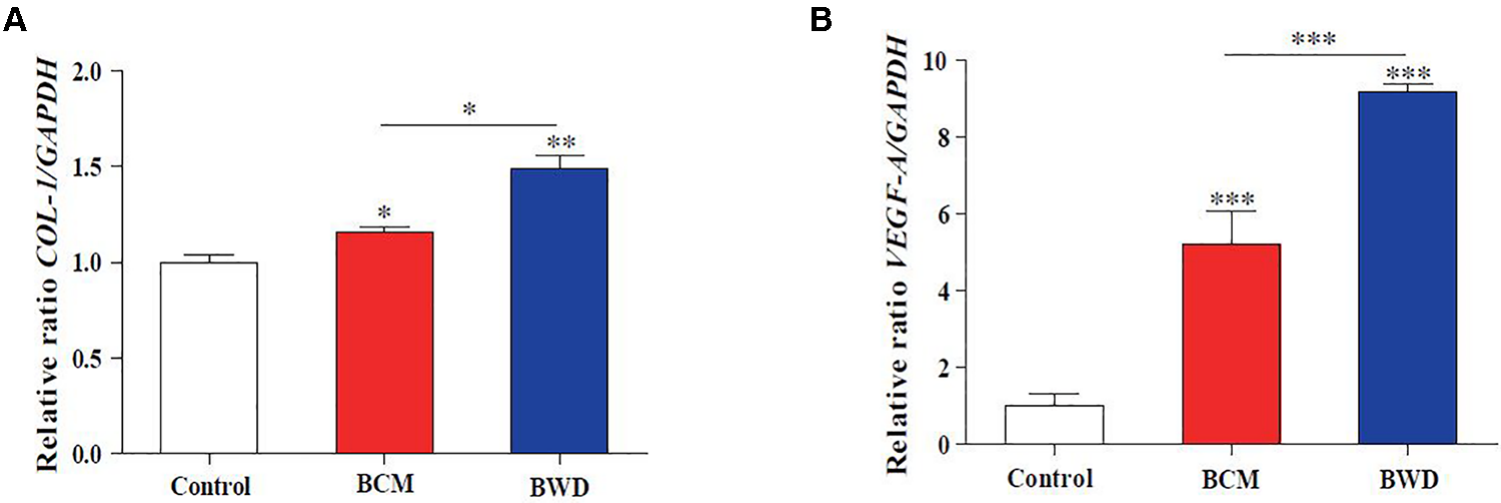
BWDs upregulate the expression of *COL-1* and *VEGF-A in vitro*. (**A**) Expression of *COL-1* in HDFs. (**B**) Expression of *VEGF-A* in HuVECs. Control, saline gauze; BCM, bacterial cellulose membrane; BWD, bioactive wound dressing; bar scales = 100 μM. Error bars represent SEM (*n* = 3), *P ≤ 0.05, **P < 0.01, and ***P ≤ 0.001.

To further investigate the molecular biological evidence that the BCM and BWD regulate the biological behaviors of HDFs and HuVECs, we detected the expressions of *COL-1* and *VEGF-A* by qRT-PCR. The figures show that the BCM and BWD could upregulate the expressions of *COL-1* in HDFs (Figure 5A) and *VEGF-A* in HuVECs (Figure 5B). The effect of BWD was more obvious (Figures 5A,B). The results suggested that the BCM and BWD promoted extracellular matrix synthesis and angiogenesis to promote wound healing by regulating the behaviors of HDFs and HuVECs.

### 3.6. BWDs promote acute wound healing in mice

**Figure 6:**
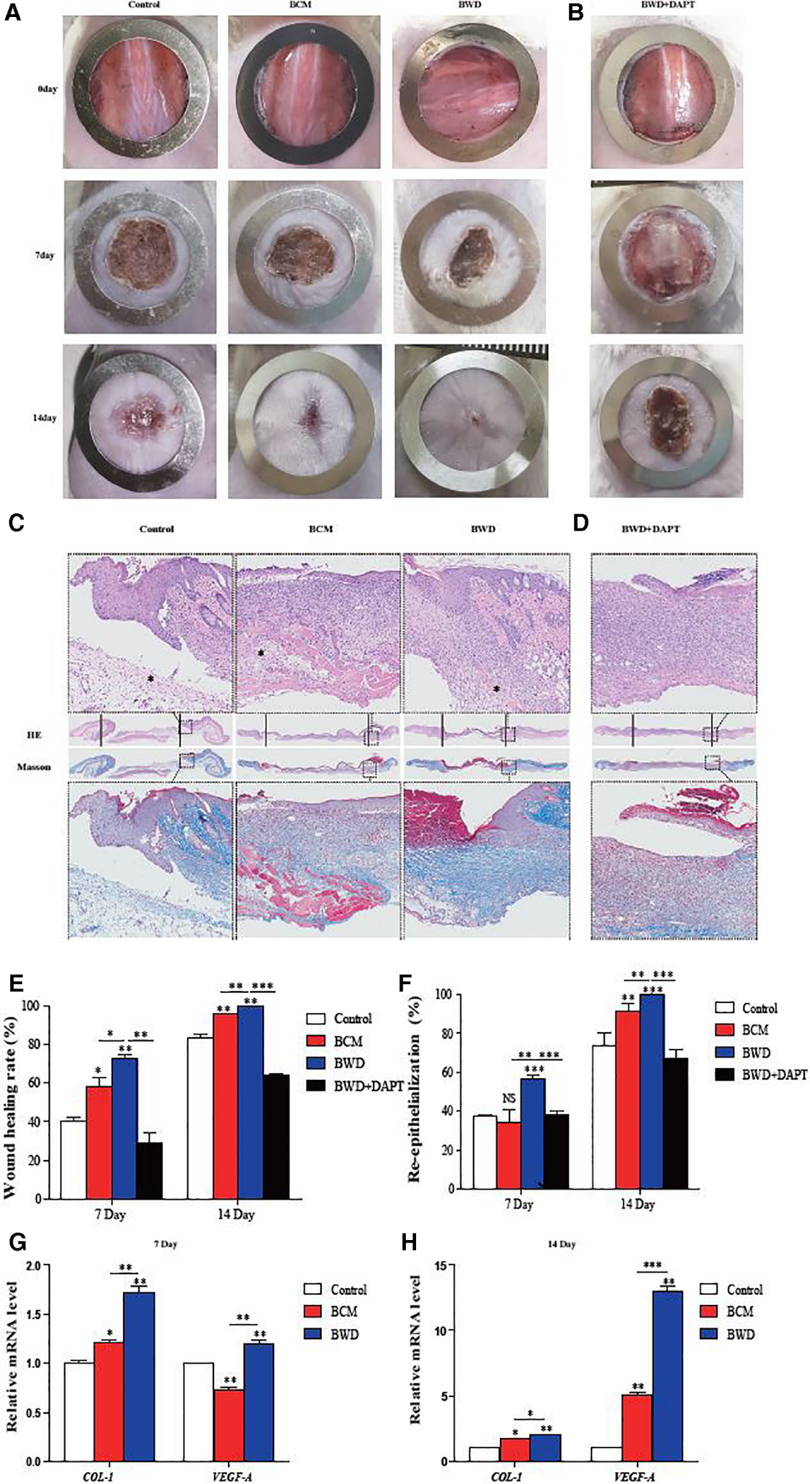
BWD promoted acute wound healing in mice. (**A**) Mouse wounds on days 0, 7, and 14 after the operation. (**B**) Mouse wounds collected on days 0, 7, and 14 after the operation, with DAPT as an inhibitor of the Notch signaling pathway. (**C**) Hematoxylin and eosin (HE) staining and Masson staining of the wound skin on day 7 (×40; * neovascularization). (**D**) HE staining and Masson staining of the wound skin on day 7, with DAPT as an inhibitor of the Notch signaling pathway (×40). (**E**) Analysis of the wound healing rate. (**F**) Statistical analysis of the epidermal gaps in each group. (**G,H**) Expressions of *COL-1* and *VEGF-A* in the wound surface detected by qRT-PCR on days 7 and 14. Error bars represent SEM (*n* = 5), *P ≤ 0.05, **P < 0.01, and ***P ≤ 0.001.

Gauze soaked with physiological saline, BCM, and BWD were used to interfere with full-thickness skin defect wounds in mice. The results showed that the wounds treated with BCM and BWD healed basically on day 14, and the effect of BWD was more significant (Figures 6A,E). The full-thickness skin of the wound was removed, fixed, dehydrated, and embedded into paraffin sections on day 7. Hematoxylin and eosin staining and Masson staining were performed to observe the angiogenesis and collagen formation. As shown in Figure 6C, compared with the control and BCM groups, the BWD group showed numerous closely arranged fibroblasts, abundant and orderly arranged collagen fibers, and more new capillaries and hair follicles. The re-epithelialization rates of all groups are presented in Figure 6F. The epidermal gaps were smaller in the BWD group on days 7 and 14 after wounding, which suggested more rapid re-epithelialization. The results showed that BWD could significantly improve wound healing.

To determine this conclusion from the molecular level, the wound samples of mice were taken on days 7 and 14, and the expressions of *COL-1* and *VEGF-A* were detected by qRT-PCR. As shown in Figures 6G,H, both BCM and BWD upregulated the expression of *COL-1* in the wound on days 7 and 14, and the effect of BWD was more obvious. Regarding *VEGF-A*, the BCM downregulated its expression on day 7, but the expression of *VEGF-A* was reversed and upregulated significantly on day 14. By contrast, the expression of *VEGF-A* in BWD-treated wounds was always at a high level. Overall, these results indicate that both the BCM and BWD could promote the healing of acute full-thickness skin defects in mice, and the effect of BWD was more significant, which was confirmed at the molecular level.

### 3.7. Activation of the notch signaling pathway

**Figure 7:**
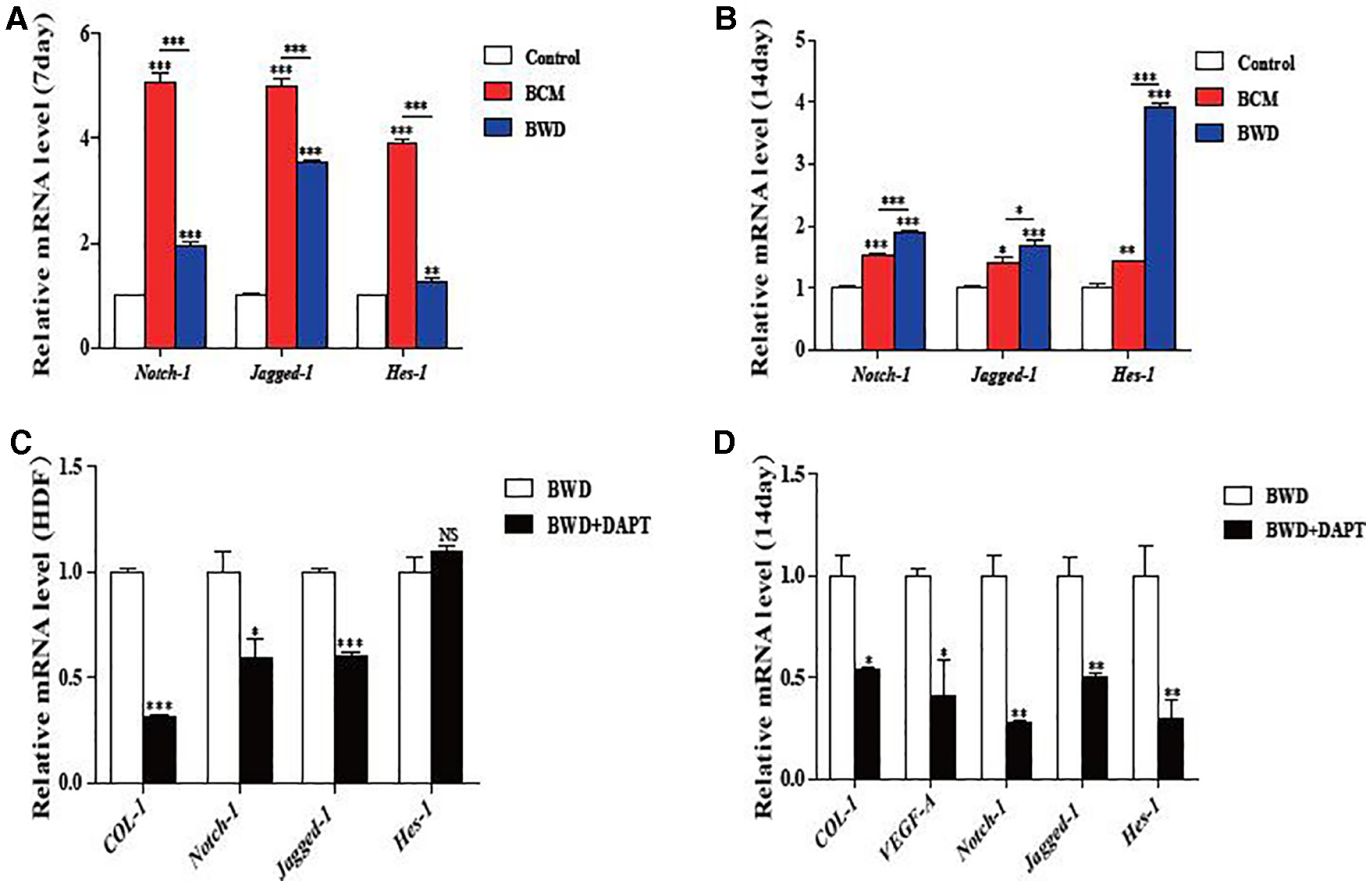
Expressions of genes in the notch signaling pathway. (**A**) Expression of genes in the Notch signaling pathway in the wound surface detected by qRT-PCR on day 7. (**B**) Expression of genes in the Notch signaling pathway in the wound surface detected by qRT-PCR on day 14. (**C**) Expressions of the Notch signaling pathway-related genes and *COL-1* in HDFs after treatment with the Notch signaling pathway inhibitor DAPT detected by qRT-PCR. (**D**) Expression of Notch signaling pathway-related genes, *COL-1* and *VEGF-A*, in the wound after treatment with the Notch signaling pathway inhibitor DAPT detected by qRT-PCR. Error bars represent SEM (*n* = 5). NS, no significant difference, *P ≤ 0.05, **P < 0.01, ***P ≤ 0.001.

The Notch signaling pathway is involved in ontogeny and tissue regeneration. We speculated that the promotion of wound healing by the BCM and BWD was related to the activation of the Notch signaling pathway. Jagged-1 is an important ligand of the Notch signaling pathway, and Hes-1 is the downstream protein. To further determine this hypothesis, wound samples of mice were taken on days 7 and 14, and the expressions of genes related to the Notch signaling pathway were detected by qRT-PCR. The expressions of *Notch-1*, *Jagged-1,* and *Hes-1* in the BCM group increased significantly on day 7 and decreased sharply on day 14. By contrast, the gene expression in the BWD group was stable at high levels (Figures 7A,B), which provided important insights that BWDs may promote wound healing by activating the Notch signaling pathway.

To determine whether BWD can promote wound healing in a Notch-dependent manner, we treated HDFs with DAPT, the *γ*-secretase inhibitor, to block the Notch receptor cleavage at the cell surface. HDFs were treated with DMSO or DAPT (5 μM), and the expression of genes related to the Notch signaling pathway and *COL-1* were detected by qRT-PCR. We found that DAPT partly abolished the positive regulating effect on *COL-1*, genes related to the Notch signaling pathway (*Notch-1* and *Jagged-1*), and expression of BWD on HDFs (Figure 7C).

Correspondingly, we performed DAPT-inhibition experiments in mice. Full-thickness skin wounds in mice were treated with BWD and DMSO or DAPT (10 μM). Pictures of wounds were collected on days 0, 7, and 14 after the operation, which also showed that DAPT partly abolished the positive regulating effect on wound healing of BWD (Figure 6B). HE staining and Masson staining were performed to observe the angiogenesis and collagen formation on day 7. BWD with DAPT showed a significant reduction of collagen fibers and angiogenesis (Figure 6D). We further verify this conclusion at the molecular level. The levels of *Notch-1*, *Jagged-1*, and *Hes-1* decreased after adding the Notch inhibitor, DAPT, indicating that DAPT successfully inhibited the Notch signaling pathway. In addition, the expressions of *COL-1* and *VEGF-A* were reduced after DAPT was added (Figure 7D). These results indicate that BWD can activate the wound healing capacity *via* the Notch signaling pathway, and these effects can be inhibited when DAPT was used, illustrating the role of the Notch signaling pathway in wound healing.

## 4. Discussion

Cellulose is one of the most abundant polymers on Earth, and it is also a valuable renewable natural resource. Cellulose includes plant cellulose (PC) and bacterial cellulose (BC). While PC is the main component of plants, BC is the primary metabolite of microorganisms, which plays a protective role. BC is synthesized by bacteria such as Acetobacter, Rhizobium, Agrobacterium, and Sarcina. BC and PC have the same chemical structural unit, which is a macromolecular polymer formed by the linkage of pyran-type glucose residues with *β*-1,4 glycosidic bond. The macrostructure and some characteristics of BC are very different from those of PC. BC has high crystallinity, polymerization degree, and chemical purity and does not contain hemicellulose, lignin, and other miscellaneous polysaccharides that are difficult to remove in plant fibers; thus, it has broad application prospects in papermaking, textile, cosmetics, medical, and other fields (23).

BCMs are considered a promising biomedical material, which can be used in drug carrier systems, tissue engineering, wound dressings, vascular implants, artificial blood vessels, biofilms, and biosensors (1–4). Its structure is similar to the extracellular matrix (such as collagen), and it can interact with biological tissues in a complex way; thus, it has a high degree of biocompatibility. In this experiment, the BCM was fermented by *Acetobacter xylinum* from Nameide Biotechnology of Shandong, China. The morphological and chemical characterization (Figure 1) and biocompatibility (Figures 2, 3) of the BCM were verified by an *in vitro* study. The effect of the BCM on wound healing was verified by *in vivo* experiments (Figures 6A,E), which are consistent with the conclusions of previous studies (18, 24).

As “seed cells” in the field of tissue engineering, BMSCs play an important role in promoting tissue repair and have been widely used in regenerative medicine and tissue repair. For skin injury, some technical difficulties are reported in transplanting BMSCs (such as low survival rate); thus, it is necessary to provide a three-dimensional space and microenvironment for “seed cells” with the help of some biological materials. In view of the good biocompatibility of the BCM and its important role in promoting wound healing, this experiment creatively combined the two by multi-point injection to prepare BWDs (Figure 3A). Furthermore, the effect of BWDs on promoting wound healing was explored, and BWDs were found to enhance the effect of the BCM (Figures 4–6). The combination of the BCM and BMSCs realized the complementary advantages and further enhanced the application effect. However, some studies have reported that MSC transplantation may lead to dangerous shortcomings, such as the possible formation of teratoma. To avoid this possible safety problem, researchers focused on BMSC-CM. It contains cytokines or mediators secreted by BMSCs to develop a cell-free therapy in stem cell therapy (25). In the *in vitro* experiment, HDFs and HuVECs were treated with the extract/CM of BWD. The extract/CM was found to promote their biological behaviors such as proliferation, migration, and extracellular matrix synthesis (Figures 4, 5). These results suggested that the active components in the extract/CM played an important role in promoting wound healing. In our experiment, BMSC was used instead of BMSC-CM combined with the BCM to prepare BWD for sustained release of cell active substances. As many studies have shown that MSCs have no short-term risk of tumorigenesis *in vivo* and *in vitro*, no tumor was found in the dose of BMSC used in this study. This proves the safe clinical application of BMSC, and it can be used as seed cells to promote wound healing in tissue engineering.

Wound healing is closely related to type I collagen and angiogenesis. HDFs participate in wound repair and tissue remodeling by proliferating and migrating to the wound and synthesizing the extracellular matrix (26). VEGF-A is a cytokine mainly derived from vascular endothelial cells, which has many functions, such as increasing vascular permeability, inducing angiogenesis and endothelial cell growth, promoting cell migration, and inhibiting apoptosis. Therefore, it plays an important role in tissue repair and wound healing (27). We proved that BWDs could promote the synthesis of the extracellular matrix and angiogenesis by upregulating the expressions of *COL-1* and *VEGF-A in vitro* (Figure 5) and *in vivo* (Figures 6G,H).

Studies have shown that the activation of the Notch signaling pathway can regulate the biological behaviors of various cells to maintain skin homeostasis, participate in skin self-repair and renewal, promote wound healing, and prolong the ability to promote wound closure (28). In addition, the Notch signaling pathway can play a key role in the formation and remodeling of the vascular network by coordinating endothelial behaviors in angiogenesis (29). Mammals have five ligands (delta-like-1 [Dll-1], delta-like-3 [Dll-3], delta-like-4 [Dll-4], jagged-1, and jagged-2) and four receptors (Notch1–4) in the signaling pathway. The activation of the Notch signaling pathway begins with a series of proteolysis processes, which are triggered by the binding of ligands and receptors between adjacent cells. First, a disintegrin and metalloproteases (ADAM) in the near membrane region are hydrolyzed, then the secretase in the transmembrane region, and finally Notch intracellular domain (NICD) is released from the cell membrane. NICD is transported to the nucleus and directly interacts with the transcription factor CSL to form a transcriptional activation complex to further regulate the expression of related genes (*bHLH, Hes, Hey/HRT/HERP,* and *Nrarp*). The expression of Notch receptors Notch-1 and Notch-4 and their ligands Jagged-1, Jagged-2, Dll-1, and Dll-4 in vascular endothelial cells *in situ* can enhance the proliferation, migration, and tube formation of vascular endothelial cells (28, 30). Evidence shows that VEGF, as an upstream regulator of the Notch signaling pathway, promotes angiogenesis by activating this pathway (31). Previous studies have shown that the regulation of this pathway can regulate the expression of angiogenic factors in vascular endothelial cells and promote angiogenesis (32). Jagged1, Dll-1, Dll-4, Notch1, and Notch4 are expressed in vascular endothelial cells. Jagged1 is a key regulator of normal angiogenesis (33), and it is widely expressed in endothelial cells during angiogenesis, especially in apical cells. It can regulate the expression of VEGF-R3 by directly transmitting Jagged1–Notch signal to apical cells to promote angiogenesis. Cutting off the Jagged1/Jagged2-mediated Notch signaling pathway inhibits tumor angiogenesis. Notch1 receptor is the most important Notch receptor in the vascular system, and its key role in coordinating angiogenesis has been widely recognized. CRISPR/Cas9-mediated Notch1 knockout inhibits the proliferation and angiogenesis of glioblastoma multiforme cells. In addition, the Notch signaling pathway activates fibroblasts for sustained collagen synthesis (34, 35). Type I collagen is the major component of the extracellular matrix. Type I collagen is a heterotrimer of col1a1 and col1a2 subunits. In fibroblasts or myofibroblasts, the two collagen polypeptides are encoded by type I collagen A1 and A2 genes (*COL1A1 and COL1A2*, respectively). Our results confirmed that BWDs could promote type I collagen synthesis and angiogenesis by upregulating the expression of wound healing-related genes (*COL-1* and *VEGF-A*) *in vivo* (Figure 5) and *in vitro* (Figures 6G,H). To further examine the mechanism, the activation of the Notch signaling pathway in wound samples showed that the gene expression of the Notch signaling pathway in the BWD group remained stable at high levels for 14 days (Figure 7). DAPT partly abolished the positive regulating effect of BWD *in vivo* and *in vitro* (Figures 6, 7). This hinted that BMSCs on the BCM can stably activate the Notch signaling pathway and provided important insights that BWDs may promote wound healing by activating the Notch signaling pathway. Some researchers have reported that BC is high-purity nanocellulose. Under certain conditions (such as pH < 7 or enzyme catalysis), it will degrade into small molecules such as monosaccharides, making it a high-quality raw material for biomedical dressings and human tissues and organs. Significant differences were also found in transcriptomics and proteomics phenotypes between HUVECs on the BCM and plastic dishes (36). These differences may be related to small molecules produced by BCM degradation. We will further explore the mechanism and target of the BCM and BWD in regulating the Notch signaling pathway.

## 5. Conclusion

This study aimed to creatively explore the effect of the BCM combined with BMSCs on wound healing. The results demonstrated that BWDs could promote the wound healing capacities of HDFs and HuVECs *in vitro* and accelerate wound healing *in vivo*. This effect may be related to the activation of the Notch signaling pathway. Our findings provided theoretical support for the clinical application of the BCM, and innovative research and production of BWDs will provide a new aspect for the treatment of skin wounds.

## Data Availability

The raw data supporting the conclusions of this article will be made available by the authors, without undue reservation.
